# Technical Note: Investigating of dosimetric leaf gap and leaf transmission factor variations across gantry and collimator angles in volumetric modulation arc therapy

**DOI:** 10.1002/acm2.14523

**Published:** 2024-09-11

**Authors:** Aram Rostami, Mojtaba Barzegar, Muhammad Usman, Satheesh Prasad Paloor, Abbass Yousef Mkanna, Alla Fuad Al‐Sabahi, Rabih Wafiq Hammoud

**Affiliations:** ^1^ Radiation Oncology Department National Center for Cancer Care and Research Doha Qatar; ^2^ Society for Brain Mapping and Therapeutic Los Angles California USA; ^3^ Intelligent Quantitative Bio‐Medical Imaging (IQBMI) Tehran Iran

**Keywords:** DLG, gantry and collimator, LTF, MLC

## Abstract

**Purpose:**

This study investigates the influence of gantry and collimator angles on the dosimetric leaf gap (DLG) and leaf transmission factor (LTF) in a Varian LINAC equipped with rounded‐end multi‐leaf collimators (MLCs). While Varian guidelines recommend DLG measurements at zero degrees for both gantry and collimator, this research aims to address the knowledge gap by assessing DLG and LTF variations at different gantry and collimator angles.

**Methods:**

Measurements were conducted using a Varian TrueBeam LINAC with a Millennium 120‐leaf MLC and Eclipse TPS version 16.1. The beams utilized in this study had energies of 6 MV, 10 MV, 6 FFF, and 10 FFF. LTF and DLG were determined using ionization chambers in solid water phantoms at various gantry angles (0°, 45°, 90°, 135°, 180°, 225°, 270°, and 315°). For each gantry angle, measurements were also taken at various collimator angles (0°, 45°, 90°, and 315°). Dosimetric impacts were evaluated through VMAT Picket Fence tests and patient‐specific verification using portal dosimetry for 10 clinical VMAT plans.

**Results:**

LTF values showed no significant variation across gantry and collimator angles. However, DLG values exhibited notable differences depending on the gantry angle and were independent of the collimator angle. The highest DLG value was observed at a gantry angle of 270 degrees, while the lowest was at 90 degrees. The AXB DLG_Average_ (averaging seven measurements of DLGs at different gantry angles) model demonstrated the best agreement between measured and calculated dose distributions, indicating the importance of considering averaged DLG values across multiple gantry angles for accurate dose calculations.

**Conclusion:**

Our study highlights the variability of DLG with gantry angle alterations, contrary to Varian guidelines recommending DLG measurements at zero gantry angle only. We advocate for utilizing an averaged DLG value from measurements across multiple gantry angles, as outlined in our methodology.

## 1 INTRODUCTION

In the domain of radiation therapy, multi‐leaf collimator (MLC)‐integrated accelerators play a pivotal role in delivering precise radiation doses to patients.^1^ The MLC is a crucial component in advanced radiation therapy techniques, particularly in intensity‐modulated radiation therapy (IMRT) and volumetric‐modulated arc therapy (VMAT).^2^, ^3^ These techniques offer significant advantages over conventional treatments by minimizing radiation exposure to healthy tissues.^4^

Accurate MLC modeling is an essential element of advanced radiation therapy technique commissioning.^5^ It facilitates the optimization of patient‐specific treatment plans, enhances the precision of radiation beam delivery, and minimizes radiation exposure to healthy tissues.^6^, ^7^, ^8^ Various MLC modeling techniques are employed across different treatment planning systems (TPS), including geometric modeling, Monte Carlo simulations, dynamic MLC modeling, dosimetric leaf gap (DLG) considerations, leaf transmission factor (LTF), leakage modeling, and advanced sequencing algorithms.^9^, ^10^

It's important to acknowledge that linear accelerators (LINACs) may exhibit variations in MLC parameters due to differences in design, technology, and specifications among various LINAC models. These disparities encompass MLC leaf design, materials used, leaf end shapes, speed, control mechanisms, and additional parameters, such as leaf penumbra, over‐travel, and interdigitating.^11^, ^12^, ^13^ The choice of leaf end shape, whether rectangular or rounded, introduces an additional layer of flexibility and precision to radiation therapy. Rounded leaf ends enhance dosimetric characteristics, such as maintaining a consistent penumbra at the isocenter plane across the treatment field. However, this also results in rounded leaf end transmission, introducing complexity into beam modeling and significantly impacting dose calculations in model‐based TPS.^14^

Two critical factors contributing to the precision modeling of rounded‐end MLCs are DLG and LTF.^13^ DLG, plays a vital role in accounting for the transmission effects of rounded leaf ends, particularly in IMRT and VMAT techniques.^2^ Simultaneously, the LTF is determined by measuring the dose with and without the shielding effect of the MLC leaves in an open field setting.

Varian accelerators utilize MLCs with rounded ends and the dose calculation algorithms of the Eclipse TPS, models the leaf ends as rectangular instead of rounded.^15^ To address this modeling discrepancy, the DLG parameter is introduced and further refined with the application of LTF. The dose calculation algorithm of the Eclipse (Varian Medical Systems Inc., Palo Alto, CA) TPS, which models the leaf ends as rectangular instead of round, incorporates the DLG parameter to account for leaf transmission by adjusting the positions of the leaf tips during fluence calculation.^2^ Each leaf tip of the MLC leaf pair is shifted back by half the DLG value, ensuring that the separation between a fully closed leaf pair matches the DLG value.^16^

Varian (Varian Medical Systems Inc., Palo Alto, CA) guidelines typically specify that DLG measurements are conducted using sweeping gap fields with both the gantry and collimator set to zero degree.^17^ However, despite numerous studies investigating the impact of gravity and mechanical sagging on MLC performance during dose delivery,^18^, ^19^ vendor recommendations lack explicit instructions for measuring the DLG and LTF at alternative gantry and collimator angles. This concern becomes especially relevant when considering that treatment techniques such as VMAT often involve gantry rotation and various gantry and collimator angles.

This study aims to address this knowledge gap by assessing the effect of gravity on variations in the value of LTF and DLG factors at different gantry and collimator angles in Varain LINAC's MLC. It will compare these values to measurements taken at zero gantry angles and investigate the implications of these variations on MLC‐based treatment plans, such as VMAT, using electronic portal imaging devices.

## 2 METHODS

### 2.1 Linac, MLC, TPS, and measurement devices

All measurements were conducted using a Varian TrueBeam machine (Varian Medical Systems, Palo Alto, CA) equipped with a Millennium 120‐leaf MLC. The beams utilized energies of 6 MV, 10 MV, 6 FFF, and 10 FFF, dose calculations were performed using an Acuros XB (AXB) algorithm within Eclipse TPS version 16.1. For dose measurements, a plastic solid water phantom (PlasticWater, CIRS), a Semiflex‐3D ionization chamber (PTW TN30021, Freiburg, Germany), and a Farmer chamber (PTW TN30021, Freiburg, Germany) were employed. Before getting any measurement in this study, dosimetic and mechanical test of linac has been done and passed by Machine Performance Check (MPC). MPC is an automated and integrated image‐based tool for verifying the beam and geometric performance of the TrueBeam linac.

### 2.2 Measurement setups

#### 2.2.1 Determination of LTF in different gantry and collimator angles

The measurement of the LTF was conducted using the source axis distance (SAD) technique with a source‐to‐surface distance (SSD) of 90 cm, at a depth of 10 cm within a solid water phantom, when the gantry was set to zero degrees. For this study, we used a Farmer chamber (TN30013) for measurements. With a length of 2.3 cm, this chamber enables the detection of intraleaf leakage from approximately four leaves, as well as five interleaf leakages, at the isocenter distance. The Farmer chamber was positioned at the isocenter depth (10 cm) with its axis oriented in the in‐plane direction.

The field size was set to 10 × 10 cm^2^, and the chamber reading was recorded as the open field reading (*R*_open_). In the next step, Farmer chamber readings were taken with the field fully blocked by Bank A, and the chamber reading was recorded as the leaf transmission for Bank A (*R_T,A_*). The same setup and procedure were repeated for Bank B, resulting in the leaf transmission for Bank B (*R_T,B_*). Finally, the average transmission (*R_T_*) was calculated as follows:

(1) $$R_{T}=\left(\frac{R_{T,A}+R_{T,B\;}}{2}\right)$$

The LTF was determined using the following equation:

(2) $$\mathrm{LTF}\;=\;\left(\frac{R_{T}}{R_{\mathrm{open}}\;}\right)$$

LTF measurement was conducted using two different approaches. In the first approach, the measurement was carried out using the SAD technique with a SSD of 90 cm, at a depth of 10 cm within a solid water phantom, with both the gantry and collimator set to zero degrees, as recommended by the vendor, and we refer to this as LTF_C0,G0_. In the second approach, LTF was measured at seven additional gantry angles (45, 90, 135, 180, 225, 270, and 315 degrees) using a setup of LTF_C0_,_G0_, without any change in the position and direction of the solid water phantom. For each gantry angle, measurements were taken at four different collimator angles: 0, 45, 90, and 315 degrees (same setup as LTF_C0_,_G0_). The final LTF calculation was obtained by averaging these seven measurements at different gantry angles, and it is referred to as LTF_Average_.

#### 2.2.2 Determination of DLG in different gantry and collimator angles

The measurement of DLG was conducted using the SAD technique with a SSD of 90 cm, at a depth of 10 cm within a solid water phantom, when the gantry was set to zero degrees. In this study, we employed a Semiflex‐3D for all DLG measurements. Positioned at the isocenter, the chamber's axis was oriented perpendicular to the direction of leaf movement. The procedure for *R_T_* measurement, as described in the LFT measurement section, was repeated and recorded using the Semiflex‐3D chamber, termed as $R_{T,DLG}$.

In the next step, readings from the Semiflex‐3D for seven different sweeping gap fields, which are moving from left to right, including 2, 4, 6, 10, 14, 16, and 20 mm, were taken and recorded as $R_{g}$. It's important to note that a 120 mm leaf travel distance was used for all gap fields. The contribution of the average MLC leaf transmission to the gap reading ($R_{gT}$) for each gap size (*g*) was calculated using the following equation:

(3) $$R_{gT}=R_{T,DLG}\;\left(1-\frac{g\left[\mathrm{mm}\right]}{120\left[\mathrm{mm}\right]}\right)$$

The corrected gap reading was then calculated for each gap size (*g*), defined as:

(4) $$R_{g^{\prime }}=R_{g}\;-R_{gT}$$

A linear function, *g* = *aR_g_*_'_ + *b*, was fitted to the points determined by the gap size (*g*) and the corrected gap reading *R_g'_*. The intercept of the fitted function (*b*) was recorded. The absolute value of “*b*” represents the DLG.

DLG measurement was conducted using two different approaches. In the first approach, the measurement was carried out using the SAD technique with a SSD of 90 cm, at a depth of 10 cm within a solid water phantom with both the gantry and collimator set to zero degrees, as recommended by the vendor, and we refer to this as DLG_C0G0_. In the second approach, DLG was measured at seven additional gantry angles (45, 90, 135, 180, 225, 270, and 315 degrees) using a setup of DLG_C0_,_G0_, without any change in the position and direction of the solid water phantom. For each gantry angle, measurements were taken at four different collimator angles: 0, 45, 90, and 315 degrees (same setup as DLG_C0_,_G0_). The final DLG calculation was obtained by averaging these seven measurements at different gantry angles which is referred to as DLG_Average_.

### 2.3 Evaluation of dosimetric impact of the DLG in different gantry angles

To assess the impact of DLG values at different gantry angles (with the collimator set to zero for all), we set up nine AXB calculation models for each photon beam, including 6 MV, 10 MV, 6 FFF, and 10 FFF. The models, denoted as AXB DLG_Average_, AXB DLG_G0_, AXB DLG_G45_, AXB DLG_G90_, AXB DLG_G135_, AXB DLG_G180_, AXB DLG_G225_, AXB DLG_G270_, and AXB DLG_G315_ utilizes the DLG _Average_, DLG_G0,_ DLG_G45_, DLG_G90_, DLG_G135_, DLG_G180_, DLG_G225_, DLG_G270_, and DLG_G315_, respectively.

In the initial phase of investigating the dosimetric effects of DLG at various gantry angles, we conducted a recalculated VMAT Picket Fence (PF) test. This test involved dynamically instructing the central 40 pairs of leaves (leaf numbers 11 to 50) from both the left bank (×1) and the right bank (×2) to move from left to right, pausing at every 1.5 cm interval to create ten gaps measuring 0.2 cm × 20 cm each. The beam was delivered during an arc rotation spanning from 179 to 187 degrees, with consistent dose rate, leaf speed, and gantry rotation speed maintained throughout the arc rotation. This setup provided direct insight into the effect of DLG values on the agreement between measured and planned MLC tests. Images were acquired using an aSi‐1200 EPID, attached to the gantry via a robotic arm, with an active area for dosimetry mode of 40 × 40 cm^2^, featuring 1190 × 1190 pixel arrays and a pixel pitch of 0.336 mm. We compared the delivered PF test with the generated plans by the TPS for all nine models (AXB DLG_Average_, AXB DLG_G0_, AXB DLG_G45_, AXB DLG_G90_, AXB DLG_G135_, AXB DLG_G180_, AXB DLG_G225_, AXB DLG_G270_, and AXB DLG_G315_) using gamma index evaluation.

In the subsequent phase of investigating the dosimetric effects of DLG values at different gantry angles, we conducted patient‐specific verification using portal dosimetry for 10 clinical VMAT plans, including five head and neck and five pelvic plans as detailed in Table 1, representing various modulation levels with different parameters like dose rate, gantry speed and leaf speed. Each plan was recalculated nine times for each calculation model, including AXB DLG_Average_, AXB DLG_G0_, AXB DLG_G45_, AXB DLG_G90_, AXB DLG_G135_, AXB DLG_G180_, AXB DLG_G225_, AXB DLG_G270_, and AXB DLG_G315_. The delivered plan of each model was then compared with the calculated plan generated by the TPS using gamma index evaluation.

**TABLE 1:** Details of clinical VMAT plan used for investigating the dosimetric effects of DLG values at different gantry angles.

Plan ID	Treatment site	PTVs and dose prescriptions	Number of arcs	Collimator angle (°)	MUs per arc
Plan 1	Head & Neck	PTV70: 70 Gy PTV54: 54 Gy	2	30, 330	600, 620
Plan 2	Head & Neck	PTV66: 66 Gy PTV54: 54 Gy	2	45, 315	590, 610
Plan 3	Head & Neck	PTV70: 70 Gy PTV50: 50 Gy	2	60, 300	605, 625
Plan 4	Head & Neck	PTV60: 60 Gy PTV50: 50 Gy	2	20, 340	580, 600
Plan 5	Head & Neck	PTV66: 66 Gy PTV54: 54 Gy	2	10, 350	595, 615
Plan 6	Pelvic	PTV78: 78 Gy PTV54: 54 Gy	3	45, 315, 30	600, 605, 508
Plan 7	Pelvic	PTV80: 80 Gy PTV50: 50 Gy	2	30, 330	710, 730
Plan 8	Pelvic	PTV78: 78 Gy PTV50: 50 Gy	2	60, 300	690, 710
Plan 9	Pelvic	PTV75: 75 Gy PTV54: 54 Gy	2	15, 345	705, 725
Plan 10	Pelvic	PTV78: 78 Gy PTV54: 54 Gy	2	20, 340	695, 715

All measurements related to DLG and LTF were repeated three times using the same setup and configurations and an averaged value was used.

## 3 RESULTS

### 3.1 LTF values in different gantry and collimator angles

The assessment of the gantry and collimator angle's impact on the LTF factor across various angles, indicates that gantry and collimator angle do not significantly influence the LTF (*p*‐value > 0.005). The corresponding LTF values for 6 MV, 10 MV, 6 FFF, and 10 FFF photon beam are presented in Table 1.

### 3.2 DLG values in different gantry and collimator angles

The assessment of the collimator angle's impact on the DLG factor across various angles, indicates that collimator angle do not significantly influence the DLG (*p*‐value > 0.005). The corresponding DLG values in different collimator angle for 6 MV, 10 MV, 6 FFF, and 10 FFF photon beam are presented in Table 2.

**TABLE 2:** DLG and LTF values in different gantry and collimator angles for 6 MV, 6 FFF, 10 MV, and 10 FFF photon beams.

Gantry angle (°)	Collimator angle (°)	6 MV DLG (mm)	10 MV DLG (mm)	6 FFF DLG (mm)	10 FFF DLG (mm)	6 MV LTF (%)	10 MV LTF (%)	6 FFF LTF (%)	10 FFF LTF (%)
**G_0_**	**0**	1.315	1.790	1.211	1.670	1.47	1.69	1.26	1.51
	**45**	1.316	1.791	1.210	1.671	1.46	1.68	1.25	1.52
	**90**	1.315	1.789	1.212	1.671	1.47	1.69	1.24	1.51
	**315**	1.315	1.789	1.212	1.670	1.45	1.67	1.26	1.53
**G_45_**	**0**	1.299	1.771	1.196	1.654	1.46	1.67	1.25	1.50
	**45**	1.298	1.770	1.196	1.653	1.47	1.66	1.26	1.51
	**90**	1.297	1.771	1.196	1.652	1.46	1.68	1.27	1.52
	**315**	1.299	1.771	1.196	1.651	1.47	1.69	1.28	1.51
**G_90_**	**0**	1.284	1.750	1.181	1.638	1.46	1.70	1.26	1.52
	**45**	1.284	1.751	1.180	1.639	1.46	1.70	1.27	1.51
	**90**	1.284	1.752	1.181	1.638	1.45	1.69	1.28	1.50
	**315**	1.289	1.750	1.180	1.639	1.47	1.68	1.26	1.51
**G_135_**	**0**	1.324	1.790	1.216	1.674	1.47	1.69	1.25	1.51
	**45**	1.325	1.791	1.214	1.674	1.47	1.68	1.26	1.52
	**90**	1.324	1.792	1.215	1.675	1.46	1.67	1.27	1.50
	**315**	1.325	1.790	1.216	1.675	1.48	1.66	1.25	1.51
**G_180_**	**0**	1.364	1.831	1.251	1.711	1.45	1.69	1.25	1.51
	**45**	1.365	1.830	1.252	1.712	1.46	1.68	1.25	1.51
	**90**	1.364	1.831	1.253	1.711	1.45	1.66	1.26	1.52
	**315**	1.365	1.831	1.251	1.712	1.46	1.66	1.24	1.50
**G_225_**	**0**	1.397	1.871	1.281	1.753	1.47	1.65	1.27	1.51
	**45**	1.397	1.870	1.279	1.751	1.48	1.66	1.28	1.52
	**90**	1.398	1.872	1.280	1.752	1.46	1.69	1.29	1.52
	**315**	1.396	1.871	1.281	1.752	1.47	1.68	1.26	1.51
**G_270_**	**0**	1.430	1.911	1.311	1.795	1.48	1.70	1.29	1.49
	**45**	1.428	1.910	1.310	1.794	1.48	1.71	1.28	1.51
	**90**	1.431	1.911	1.323	1.794	1.49	1.69	1.28	1.52
	**315**	1.430	1.912	1.322	1.795	1.47	1.70	1.27	1.53
**G_315_**	**0**	1.371	1.850	1.261	1.732	1.47	1.68	1.25	1.54
	**45**	1.372	1.851	1.260	1.732	1.48	1.69	1.26	1.51
	**90**	1.371	1.851	1.259	1.731	1.46	1.70	1.27	1.53
	**315**	1.372	1.851	1.261	1.731	1.47	1.71	1.25	1.52

**FIGURE 1:**
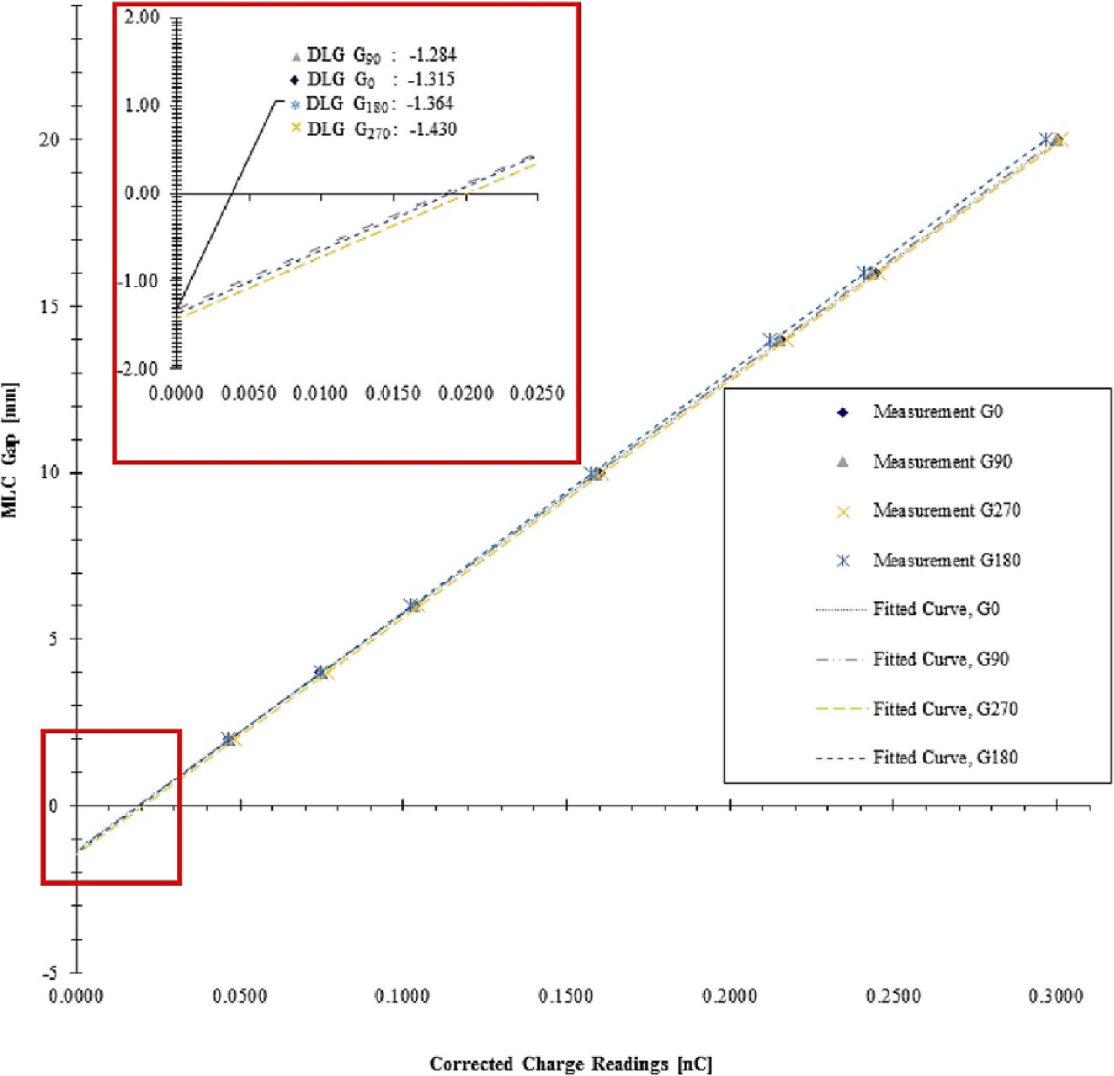
Extrapolation graph to calculate DLG for 6 MV photon beam using solid water phantom with Semiflex3D ionization chamber for gantry angles at 0 (G_0_), 90 (G_90_), 270 (G_270_), and 180 (G_180_).

On the other hand, the assessment of the gantry angle's impact on the DLG factor across various angles, indicates the impact of the gantry angle on the value of DLG for all included energies in this study (*p*‐value < 0.005). Figure 1 depicts the variation of DLGs across different gantry angles (G_0_, G_90_, G_180_, and G_270_) for a 6 MV photon beam, measured using a solid water phantom with a Semiflex‐3D ionization chamber. DLG values were determined through an extrapolation graph that correlates corrected charge readings with sweeping MLC gaps, as illustrated in Figure 1. For 6 MV photon beam, the measured DLG absolute values in climator zero angles were 1.315, 1.299, 1.284, 1.324, 1.364, 1.397, 1.43, and 1.372 mm for G_0_, G_45_, G_90_, G_135_, G_180_, G_225_, G_270_, and G_315_, respectively (*p*‐value < 0.005). The highest DLG value was observed for G_270_, while the minimum DLG value was observed for G_90_. The average calculated DLG (DLG_Average_) is 1.348 mm. DLG values in different gantry and collimator angles for 10 MV, 6 FFF, and 10 FFF are presented in Table 2.

### 3.3 Dosimetric impact of the DLG values in different gantry angles

**FIGURE 2:**
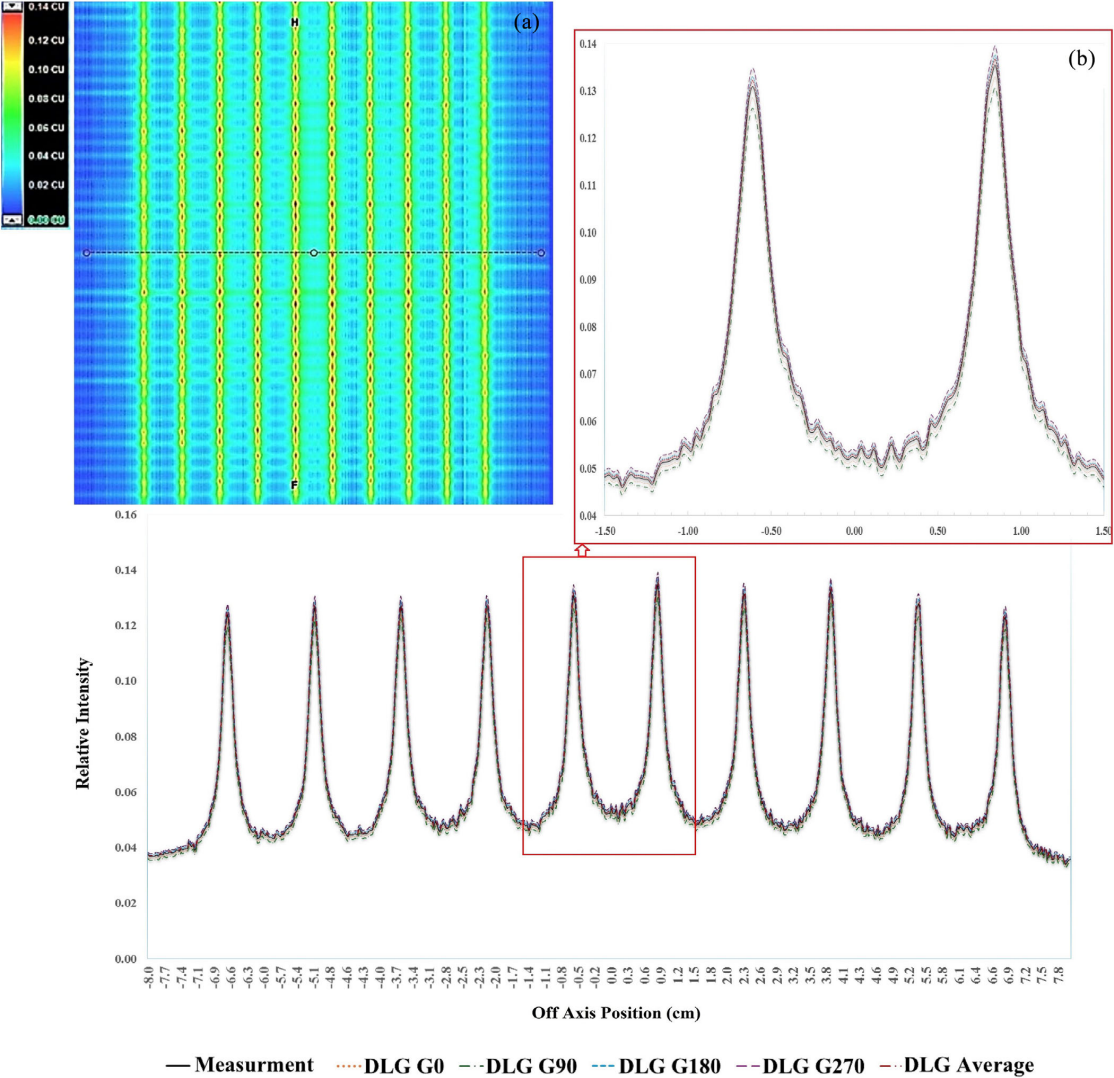
(a) Measured Picket Fence (PF) test by EPID; (b) intensity profile of PF test measured by EPID in compare with PF plans calculated by Eclipse treatment planning system for AXB DLG_Average_, AXB DLG G_0_, AXB DLG G_90_, AXB DLG G_180_, and AXB DLG G270 (6 MV Photon Beam).

In this study, the AXB dose calculation algorithm in Eclipse TPS was utilized for dose calculation. This algorithm requires the DLG value to be measured by the user and imported into the TPS for any plan that utilizes MLC. To assess the dosimetric impact of DLG at different gantry angles, for each photon beam energy (include 6 MV, 10 MV, 6 FFF, and 10 FF) AXB was supplied with various measured DLG values at G_0_, G_45,_ G_90_, G_135,_ G_180_, G_225,_ G_270_, and G_315_ and the DLG_average_ was used to create nine different AXB dose calculation algorithms fed by nine different DLG values. For each dose calculation model, including AXB DLG_Average_, AXB DLG_G0_, AXB DLG_G45_, AXB DLG_G90_, AXB DLG_G135_, AXB DLG_G180_, AXB DLG_G225_, AXB DLG_G270_, and AXB DLG_G315_, the intensity profiles of the PF plans on the central axis are compared with the intensity profile of the measured PF test by EPID (Figure 2a). Figure 2b illustrates the agreement between the measured fluence by EPID and the calculated fluence by Eclipse for AXB DLG_Average_, AXB DLG_G0,_ AXB DLG_G90,_ AXB DLG_G180,_ AXB DLG_G270_ dose calculation model for 6 MV photon beam.

**FIGURE 3:**
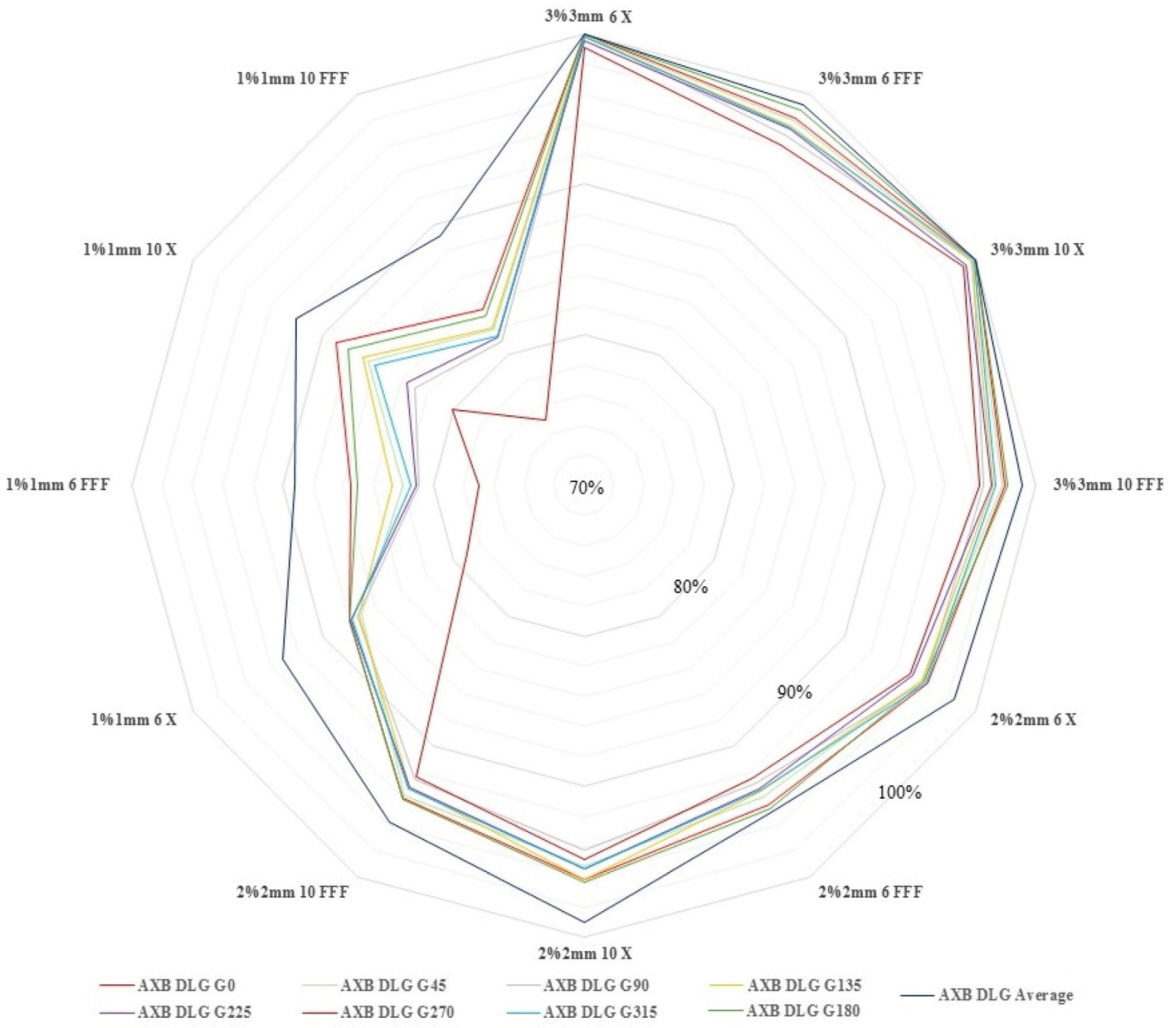
Global gamma evaluation between measured and calculated Picket Fence by Eclipse treatment planning system for AXB DLG_Average_, AXB DLG G_0_, AXB DLG G_45_, AXB DLG G_90_, AXB DLG G_135_, AXB DLG G_180_, AXB DLG G_225,_ AXB DLG G_270_, and AXB DLG G_315_: pass rate with gamma evaluation of 3%/ 3 mm, 2%/ 2 mm, and 1%/ 1 mm for 6 MV, 6 FFF, 10 MV, and 10 FFF photon energies.

Figure 3 displays the global gamma index pass rates (3%/3 mm), (2%/2 mm), and (1%/1 mm) between the measured PF test and the PF plans created in the TPS for AXB DLG_Average_, AXB DLG_G0_, AXB DLG_G45_, AXB DLG_G90_, AXB DLG_G135_, AXB DLG_G180_, AXB DLG_G225_, AXB DLG_G270_, and AXB DLG_G315_. AXB DLG_Average_ demonstrated the best agreement between the measurement and the planned PF pattern, while the worst case was observed for AXB DLG_G270_.

Table 3 shows the average gamma index pass rate of patient‐specific verification using portal dosimetry for 10 clinical VMAT plans, for each photon beam energy, including 6 MV, 10 MV, 6 FFF, and 10 FF, each plan was recalculated nine times for each calculation model, which AXB DLG_Average_, AXB DLG_G0_, AXB DLG_G45_, AXB DLG_G90_, AXB DLG_G135_, AXB DLG_G180_, AXB DLG_G225_, AXB DLG_G270_, and AXB DLG_G315_. AXB DLG_Average_ for all energies showed the highest average gamma index pass rate. Lowest pass rate seen for AXB DLG_G270,_ same for all energies.

**TABLE 3:** Average global gamma pass rate of patient‐specific verification using portal dosimetry for 6 MV, 10 MV, 6 FFF, and 10 FFF MV photon beams using nine dose calculation models include AXB DLG_Average_, AXB DLG_G0_, AXB DLG_G45_, AXB DLG_G90_, AXB DLG_G135_, AXB DLG_G180_, AXB DLG_G225_, and AXB DLG_G270_, AXB DLG_G315_ for: Pass rate with gamma evaluation of 3%, 3 mm, 2%, 2 mm, and 1%, 1 mm.

		Average global gamma pass rate (%)								
Photon energy (MV)	Gamma index (criteria)	AXB DLG_G0_	AXB DLG_G45_	AXB DLG_G90_	AXB DLG_G135_	AXB DLG_G180_	AXB DLG_G225_	AXB DLG_G270_	AXB DLG_G315_	AXB DLG_Average_
**6**	**3%/**3 mm	98.1 ± 1.9	96.5 ± 1.1	95.6 ± 2.1	96.0 ± 0.9	96.0 ± 1.9	95.0 ± 1.6	94.2 ± 2.6	96.3 ± 1.3	99.2 ± 0.8
	**2%/**2 mm	93.1 ± 2.2	92.1 ± 1.5	91.4 ± 2.5	91.8 ± 1.8	92.8 ± 1.6	90.9 ± 2.5	90.1 ± 2.1	91.8 ± 1.1	97.3 ± 1.1
	**1%/**1 mm	80.2 ± 1.1	79.5 ± 0.6	78.5 ± 1.6	78.9 ± 0.3	79.3 ± 1.3	73.9 ± 1.9	73.8 ± 1.1	79.0 ± 0.5	88.7 ± 0.6
**10**	**3%/**3 mm	97.6 ± 1.4	96.0 ± 0.6	95.1 ± 1.6	95.5 ± 0.8	95.5 ± 1.4	94.5 ± 1.2	93.7 ± 2.1	95.8 ± 1.1	98.8 ± 0.9
	**2%/**2 mm	92.6 ± 1.7	91.6 ± 1.0	90.9 ± 2.0	91.3 ± 1.3	92.3 ± 1.1	90.5 ± 2.0	89.6 ± 1.5	91.2 ± 1.9	96.7 ± 1.1
	**1%/**1 mm	79.7 ± 1.0	79.0 ± 0.9	78.0 ± 1.1	78.4 ± 0.4	78.7 ± 0.8	73.5 ± 1.4	73.3 ± .8	78.5 ± 0.5	87.7 ± 0.9
**6 FFF**	**3%/**3 mm	97.9 ± 1.0	96.1 ± 0.6	95.2 ± 2.5	95.8 ± 1.4	96.7 ± 1.1	94.8 ± 1.6	93.5 ± 2.7	96.1 ± 2.1	99.1 ± 1.9
	**2%/**2 mm	92.3 ± 1.2	92.1 ± 1.0	91.2 ± 3.0	91.3 ± 1.1	92.6 ± 1.4	90.8 ± 2.1	89.9 ± 1.1	91.5 ± 1.4	97.0 ± 1.4
	**1%/**1 mm	79.1 ± 1.9	79.2 ± 0.9	77.2 ± 2.1	78.6 ± 1.1	78.9 ± 2.1	73.7 ± 2.1	73.5 ± 1.8	78.5 ± 1.5	88.1 ± 1.2
**10 FFF**	**3%/**3 mm	97.6 ± 1.9	96.8 ± 1.1	95.3 ± 2.1	96.0 ± 0.9	96.3 ± 1.9	95.2 ± 1.6	93.2 ± 2.6	96.3 ± 1.3	99.4 ± 0.8
	**2%/**2 mm	93.2 ± 2.2	92.3 ± 1.5	91.5 ± 2.5	91.9 ± 1.8	92.7 ± 1.6	91.4 ± 2.5	90.3 ± 2.1	91.9 ± 1.1	96.1 ± 1.1
	**1%/**1 mm	79.6 ± 1.0	78.0 ± 0.9	77.0 ± 1.1	78.0 ± 0.4	78.1 ± 0.8	73.5 ± 1.4	73.1 ± .8	78.5 ± 0.5	83.1 ± 0.9

## 4 DISCUSSION

Several studies have shown the influence of gravity on the mechanical performance of linear accelerators.^18^, ^19^, ^20^, ^21^, ^22^

In the present study, the effect of gantry and collimator angle on LTF was investigated and showed no significant impact on the amount of radiation passing through the leaves. Similarly, no significant impact of the collimator angle was observed for DLG values. However, there were variations when considering the effect of gantry angle on DLG, likely due to gravitational effects. The standout finding was that the highest DLG value occurred when the gantry angle was at 270 degrees, which is when the MLCs are moving against gravity. Okumura et al.^23^ revealed that the minimum leaf velocity of Varian LINACs was observed at a gantry angle of 270 degrees, corresponding to a situation where the leaves are traveling opposite to the gravitational force, possibly leading to prolonged leaf transit times. Our results are in line with this study. Additionally, the results of our study showed the potential for reducing DLG values when the gantry angle is at 90 degrees, where the MLCs are moving in alignment with gravity. Evaluation of DLG values across different gantry angles for various photon beam energies (6 MV, 10 MV, 6 FFF, and 10 FFF) showed the same changes for all energies, indicating energy independence (see Table 1)

As mentioned, our results showed that the highest DLG value was observed at a gantry angle of 270 degrees and the lowest at 90 degrees, which we attributed to the effect of gravity. The key aspect to understand here is the complex interplay between the gantry and collimator angles and their combined effect on MLC motion and DLG. The collimator angle was found to have a negligible influence on the DLG and LTF, even at 90 and 270 degrees for all gantry angles. This outcome is likely because the collimator angle primarily adjusts the orientation of the MLC leaves but does not significantly affect the force exerted by gravity on the MLC leaves themselves. Another justification could be that the mechanical design and control of the MLC system are such that the gravitational impact on leaf motion is predominantly influenced by the gantry angle rather than the collimator angle.

In our investigation of dosimetric effects of DLG values for different gantry angles, the creation of multiple calculation models includes AXB DLG_Average_, AXB DLG_G0_, AXB DLG_G45_, AXB DLG_G90_, AXB DLG_G135_, AXB DLG_G180_, AXB DLG_G225_, AXB DLG_G270_, and AXB DLG_G315_, allowed for comprehensive evaluation across various gantry angles. Notably, the AXB DLG_Average_ model, incorporating averaged DLG values from measurements across multiple gantry angles, emerged as the most robust in terms of agreement between measured and calculated dose distributions.

Looking at the percentage gamma pass rate for PF test and patient's VMAT plans (Figure 3 and Table 2), the scoring of dose calculation algorithms can be taken as follows (from best to worst): AXB DLG_Average_, AXB DLG_G0_, AXB DLG_G180_, AXB DLG_G90_, and AXB DLG_G270_ for all evaluated photon beams including 6 MV, 10 MV, 6 FFF, and 10 FFF.

Although the Varian guideline recommends measuring DLG and LTF at G_0_ degrees without addressing different gantry angles,^24^ our study demonstrates the variability of DLG with gantry angle alterations. Consequently, utilizing an averaged DLG value from measurements across multiple gantry angles, as outlined in our methodology, is advocated.

These findings underscore the importance of considering mechanical factors, particularly gantry angle effects, in MLC modeling and treatment planning. DLG variations can directly impact dose calculations and beam modulation, posing challenges to treatment efficacy and patient safety. Therefore, understanding and mitigating the impact of gravity‐induced mechanical deformations on DLG are crucial for ensuring precise and reliable radiation therapy delivery. Future research should explore the generalizability of our findings to other LINAC models and MLC designs, especially in Elekta and Tomotherapy machines, which can perform VMAT treatments, where the gantry rotation can be affected by gravity with respect to the MLC and other mechanical parameters. Varian, Elekta, and Tomotherapy systems have differences in MLC positioning, MLC end design, and MLC speed. These differences impact the dependency of MLC performance at different gantry angles.

## 5 CONCLUSION

Our study highlights the variability of DLG with gantry angle alterations, contrary to Varian guidelines recommending DLG measurements at G_0_ degrees only. Since Eclipse TPS accepts only one value for DLG per energy, we advocate for utilizing an averaged DLG value from measurements across multiple gantry angles, as outlined in our methodology.

## AUTHOR CONTRIBUTIONS

Aram Rostami: Co‐authored sections, directed all content, proofing all content, contributed to running the gamma test and analysis, and contributed to writing the manuscript. Mojtaba Barzegar: Co‐authored sections, created figures, and contributed to writing the manuscript. Muhammad Usman: Contributed to DLG measurement and proofing all content. Satheesh Prasad Paloor: Co‐authored sections, directed all content, and co‐developed proofing all content. Abbass Yousef Mkanna: Contributed to DLG measurement and contributed to writing the manuscript. Alla Fuad Al‐Sabahi: Co‐authored sections and created figures. Rabih Hammoud: Co‐authored sections, directed all content, and co‐developed proofing all content.

## CONFLICT OF INTEREST STATEMENT

The authors declare no conflicts of interest.
